# Subject-specific multi-poroelastic model for exploring the risk factors associated with the early stages of Alzheimer's disease

**DOI:** 10.1098/rsfs.2017.0019

**Published:** 2017-12-15

**Authors:** Liwei Guo, John C. Vardakis, Toni Lassila, Micaela Mitolo, Nishant Ravikumar, Dean Chou, Matthias Lange, Ali Sarrami-Foroushani, Brett J. Tully, Zeike A. Taylor, Susheel Varma, Annalena Venneri, Alejandro F. Frangi, Yiannis Ventikos

**Affiliations:** 1 Department of Mechanical Engineering, University College London, London, UK; 2 Centre for Computational Imaging and Simulation Technologies in Biomedicine (CISTIB), Department of Electronic and Electrical Engineering, University of Sheffield, Sheffield, UK; 3 IRCCS San Camillo Foundation Hospital, Venice, Italy; 4 Centre for Computational Imaging and Simulation Technologies in Biomedicine (CISTIB), Department of Mechanical Engineering, University of Sheffield, Sheffield, UK; 5 Institute of Biomedical Engineering and Department of Engineering Science, University of Oxford, Oxford, UK; 6 Children's Medical Research Institute and School of Medical Sciences, Sydney Medical School, The University of Sydney, Westmead, Australia; 7 Department of Neuroscience, Medical School, University of Sheffield, Sheffield, UK

**Keywords:** Alzheimer's disease, poroelasticity, finite-element method, permeability tensor map, cerebral blood flow, cerebrospinal fluid

## Abstract

There is emerging evidence suggesting that Alzheimer's disease is a vascular disorder, caused by impaired cerebral perfusion, which may be promoted by cardiovascular risk factors that are strongly influenced by lifestyle. In order to develop an understanding of the exact nature of such a hypothesis, a biomechanical understanding of the influence of lifestyle factors is pursued. An extended poroelastic model of perfused parenchymal tissue coupled with separate workflows concerning subject-specific meshes, permeability tensor maps and cerebral blood flow variability is used. The subject-specific datasets used in the modelling of this paper were collected as part of prospective data collection. Two cases were simulated involving male, non-smokers (control and mild cognitive impairment (MCI) case) during two states of activity (high and low). Results showed a marginally reduced clearance of cerebrospinal fluid (CSF)/interstitial fluid (ISF), elevated parenchymal tissue displacement and CSF/ISF accumulation and drainage in the MCI case. The peak perfusion remained at 8 mm s^−1^ between the two cases.

## 1. Introduction

### 1.1. Dementia, mild cognitive impairment and Alzheimer's disease

Alzheimer's disease (AD) is the most common form of dementia, a clinical syndrome of progressive deterioration of cognitive abilities and ordinary daily functioning [1]. Microscopically, it is characterized by the excessive accumulation of neurotoxic amyloid-β (Aβ) into parenchymal senile plaques or within the walls of arteries and capillaries in addition to the aggregation of hyperphosphorylated *τ* into intracellular neurofibrillary tangles and neuropil threads [2,3]. Macroscopic pathological alterations include atrophy, ventriculomegaly, cortical thinning and white matter abnormalities [2,4]. In its early stage, AD may present itself as mild cognitive impairment (MCI), an intermediate state between normal ageing and dementia [5,6]. It affects 19% of people aged over 65 [7]. It is estimated that 46% of people with MCI develop dementia within 3 years, compared with 3% of the age-matched population [8].

Modelling transport of fluid within the brain, in a personalized manner and from first principles, is essential in order to help decipher some of the underlying mechanisms that are currently being investigated with regard to AD. The amyloid hypothesis has long been the main driver in trying to unravel the root causes of this disease. It has been criticized for its lack in coherent evidence, in addition to its failure in providing effective treatment regimens [9,10]. An important question that must be answered relates to whether the Aβ cascade hypothesis is the underlying cause of AD [10]. While Aβ is a key molecule in AD, there is evidence to suggest (along with genetic risk factors [11]) that AD may be a vascular disorder [12,13], caused by impaired cerebral perfusion [9,13], which is detectable even in its early prodromal MCI stage [10,14,15]. The causes of chronic hypoperfusion may be varied (e.g. [16]), but there is evidence that it might be promoted by cardiovascular risk factors [17], which in turn are strongly influenced by lifestyle [18]. Modelling perfused parenchymal tissue may enhance our understanding of the influence of modifiable lifestyle factors (LFs) such as smoking, dietary habits and leisure activities [19] in addition to environmental risk factors (such as sleep impairment, diabetes and hypertension) [2] in AD chronic hypoperfusion. This conceptual understanding is expected to provide novel biomarkers, especially in the early stages of AD.

### 1.2. Multiple-network poroelastic theory

The classical form of Biot's consolidation theory [20,21] is described for an isotropic and nearly incompressible solid matrix and homogeneous porous medium. The formulation comprises three components: a mechanical equilibrium equation governing elastic deformation; Darcy's law for modelling fluid flow; and a mass conservation expression. In the cerebral environment, the classical Biot's theory has been extended to a multiple-network poroelastic theory (MPET) formulation [22].

### 1.3. Outline of the article

This paper introduces a novel consolidated pipeline that integrates three important components: a three-dimensional (3D) MPET-based model of cerebral parenchyma; an accurate, fully automated image-based model personalization workflow; and a subject-specific boundary condition model. This pipeline is expected to provide a template in obtaining novel biomarkers during the early stages of AD. The essential breakdown of the methodology behind the full implementation scheme will follow in §2, which highlights the integrated nature of the consolidated pipeline embedded within the VPH-DARE@IT research platform, and the prospective data collection programme used to extract the subject-specific data used in this study. This section is accompanied by mesh independence studies. In §3, four MPET simulations are presented, based on one control and one MCI subject, each during high and low activity states. The results are discussed in §4, along with limitations and perspectives for future work. The conclusions to the paper are given in §5.

## 2. Methodology

### 2.1. Cross-sectional case–control study of cerebral blood flow variability

The subject-specific datasets used in the modelling of this paper were collected as part of the VPH-DARE@IT project (www.vph-dare.eu), and prospective data collection was conducted at the Istituto di Ricovero e Cura a Carattere Scientifico (IRCCS) San Camillo, Lido di Venezia, Italy (hereafter referred to as the ‘Lido study’). The Lido study, including a total of 103 people (50 cognitively healthy controls, age 71.1 ± 7.9 years, and 53 with diagnosed MCI, age 75.1 ± 6.7 years), was approved by the joint ethics committee of the Health Authority Venice 12 and the IRCCS San Camillo (Protocol number 2014.08), and all participants gave informed consent prior to participation in the study.

For each subject, several measurement modalities were collected: lifestyle questionnaires and neuropsychological tests, whole-brain magnetic resonance (MR) imaging, clinical ultrasound flow imaging, portable Holter recordings of blood pressure and actigraph measured activity levels, among others. Lifestyle information was collected by means of established questionnaires (CAIDE study [23]). Clinical ultrasound imaging comprised both carotid ultrasound and cardiac echography (Siemens Acuson X300PE and SC2000, Siemens Healthineers, Erlangen, Germany). Portable Holter devices (Cardioline walk200b, Cardioline S.p.A., Milan, Italy) measured both blood pressure and the electrocardiogram. Physical activity and sleep were measured using wrist-portable actigraph devices (MotionWatch 8, CamNtech Ltd, Cambridge, UK). For the Lido study cohort, T1-weighted (T1w) and diffusion-weighted MR images were processed to create accurate 3D whole-brain meshes and permeability tensor maps (PTMs) of the parenchyma using the workflow described in §§2.3.1 and 2.3.2; and Holter recordings and ultrasound flow measurements were used to generate boundary conditions (BCs) of arterial blood flow using the models described in §2.3.3.

In this preliminary study, two cases were analysed, one male control (66 years of age) and one male MCI case (78 years of age). Information relating to weight, smoking, daily leisure time and length of sleep was recorded. Both cases were non-smokers. The daily leisure time ranged from less than 15 min per day (control) to greater than 1 h (MCI case). The length of sleep was unavailable for the control subject, whereas for the MCI case, the recorded time was approximately 6 h.

### 2.2. Three-dimensional multi-poroelastic model for the cerebral environment

**Figure 1.**
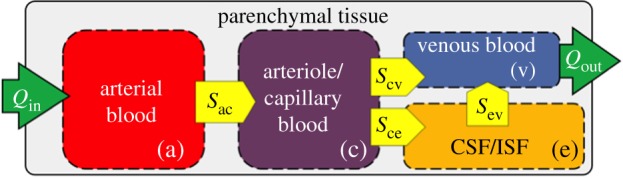
The four-compartment MPET model. Flow is prohibited between the CSF and the arterial network, while directional transfer exists between (a) and (c), (c) and (v), (c) and (e) and finally (e) and (v). (Online version in colour.)

In this paper, the MPET (or multi-poroelastic) model was used to conduct mechanistic modelling of fluid transport through the brain parenchyma. Biologically, in a porous medium representing the cerebral environment, the solid matrix represents brain parenchyma, and the communicating fluid phases taken into account are: an arterial network (a), an arteriole/capillary network (c), a cerebrospinal fluid (CSF)/interstitial fluid (ISF) network (e) and a venous network (v) (figure 1). This model allows for the simultaneous solutions of continuity and momentum conservation equations, in four interconnected fluid compartments, within a deformable solid matrix (parenchymal tissue).

#### 2.2.1. Governing equations

The MPET model uses the parenchymal tissue displacement (**u**), and the pore pressures of the four fluid compartments (*p*_a_, *p*_c_, *p*_e_, *p*_v_) as the primitive variables in the governing equations, which are listed below (equations (2.1)–(2.5)):
2.1
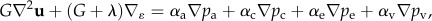

2.2
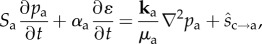

2.3
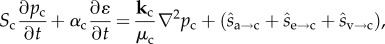

2.4
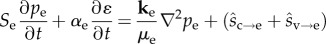

2.5
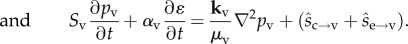


Equation (2.1) is the equilibrium equation, which describes the momentum balance in the porous medium. Here, **u** is the displacement vector; *p_i_* the scalar pore pressure in each fluid compartment (*i* = a, c, e and v); *G* the shear modulus; *λ* the Lamé's constant; *ɛ* the dilatational strain; *α_i_* the Biot–Willis coefficient for each fluid compartment which satisfies *ϕ* ≤ *α*_a_ + *α*_c_ + *α*_e_ + *α*_v_ ≤ 1 [24,25], where *ϕ* is the total porosity.

Equations (2.2)–(2.5) are continuity equations, which describe the mass balance. Here, *S_i_* is the specific storage (a measure of the released fluid volume per unit pressure in the control volume at constant strain for each fluid compartment); **k***_i_* is the permeability tensor for each of the four fluid compartments, which reduces to **k***_i_* = *k_i_***I**, with *k_i_* a constant and **I** the unit tensor, for an isotropic medium; and *µ_i_* is the viscosity of each fluid compartment. The *ŝ* terms in equation (2.2)–(2.5) define spatially varying source 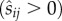 or sink 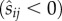 densities (rate of fluid transfer between networks) [22,26,27].

#### 2.2.2. Boundary conditions and poroelastic constants

**Figure 2.**
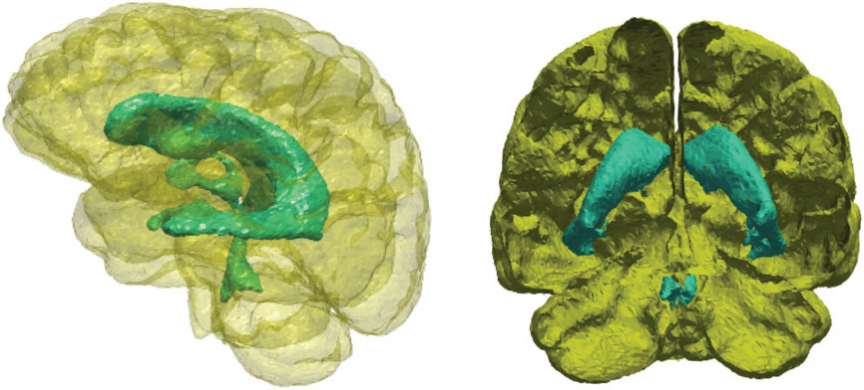
The two boundary surfaces used to define the volume of the parenchymal tissue. The outer surface (yellow) represents the cortical surface and the inner surface (turquoise) represents the ventricular wall. (Online version in colour.)

In this paper, the volumetric domain that represents the parenchymal tissue is bounded by two surfaces, the outer boundary is the cortical surface and the inner boundary is the ventricular wall (figure 2). Both surfaces are closed, and the space enclosed by the ventricular wall lies within the cortical domain. The volumetric domain used in modelling is the space between these two surfaces. The BCs for both the solid and fluid phases are listed in table 1.

**Table 1.** BCs used in the MPET modelling.

	cortical surface		ventricular wall	
displacement	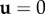	(2.6)	*no displacement constraints*	
arterial blood	*subject-specific blood flow*		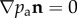	(2.7)
arteriole/capillary blood	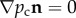	(2.8)	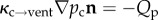	(2.9)
CSF/ISF	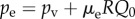	(2.10)	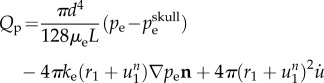	(2.11)
venous blood	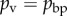	(2.12)	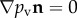	(2.13)

The skull is assumed rigid (adult cases). For simplicity, this rigid BC is applied directly to the cortical surface, which assumes no displacement is allowed at this boundary (equation (2.6)). There are no displacement constraints at the ventricular wall, so it can expand or contract freely. For continuity of stresses, the pressure exerted by the CSF within the ventricles on the inner ependymal surface must balance the poroelastic stress in the parenchymal tissue [22,26–28]. A subject-specific blood flow profile is used as the BC for the arterial network at the cortical surface (see §2.3.3). Descriptions for equations (2.7)–(2.13) can be found in previous studies [22,26]. Table 2 gives the list of parameters used in the MPET modelling framework. The majority of the parameters in this table have been used in previous studies [22,26–31]. It should be noted that the permeability of the CSF/ISF compartment, *k*_e_, listed in table 2 is the base permeability, which is multiplied by the dimensionless permeability tensor (see §2.3.2) to calculate the actual permeability for the CSF/ISF compartment in the subject-specific modelling.

**Table 2.** Parameters used in the MPET modelling.

parameters	values	units	parameters	values	units
*α*_a__,c_	0.25		*k*_a,c,e,v_	1.0 × 10^−10^	m^2^
*α*_e_	0.49		*ω*_ac_	1.5 × 10^−19^	m^2^ N^−1^s^−1^
*α*_v_	0.01		*ω*_cv_	1.5 × 10^−19^	m^2^ N^−1^s^−1^
*λ*	505	Pa	*ω*_ev_	1.0 × 10^−13^	m^2^ N^−1^s^−1^
*G*	216	Pa	*ω*_ce_	1.0 × 10^−20^	m^2^ N^−1^s^−1^
*L*	70 × 10^−3^	m	*R*	8.5 × 10^13^	m^−3^
*d*	3 × 10^−3^	m	*Q*_p_	5.8 × 10^−9^	m^3^s^−1^
*p*_bp_	650	Pa			
*S*_a__,c_	2.9 × 10^−4^	m^2^ N^−1^			
*S*_e_	3.9 × 10^−4^	m^2^ N^−1^			
*S*_v_	1.5 × 10^−5^	m^2^ N^−1^			

#### 2.2.3. Mesh independence test of multi-poroelastic brain modelling

**Figure 3.**
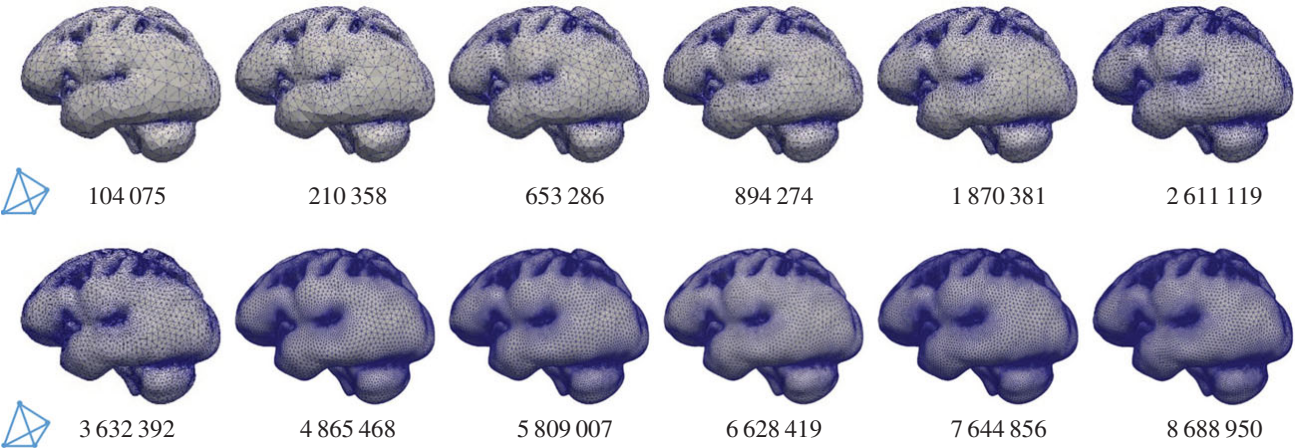
Twelve meshes with varying element numbers, discretized from the same cerebral geometry. The number of tetrahedral elements is indicated in each case. (Online version in colour.)

The highly coupled governing equations of the MPET have been discretized using the finite-element method and implemented into an in-house numerical code, which has been verified [32] against Terzaghi's [33] and Mandel's [34] problems. Here, the mesh dependence of the 3D MPET simulations using a realistic brain geometry was investigated, which provided guidance on mesh resolution in the subject-specific modelling. This geometry has similar complexity, e.g. the details of the ventricles, sulci and gyri, etc., as the ones used in the subject-specific modelling. Twelve meshes were created from the same brain geometry, with total element numbers ranging from approximately 100 000 to approximately 9 million (figure 3).

**Figure 4.**
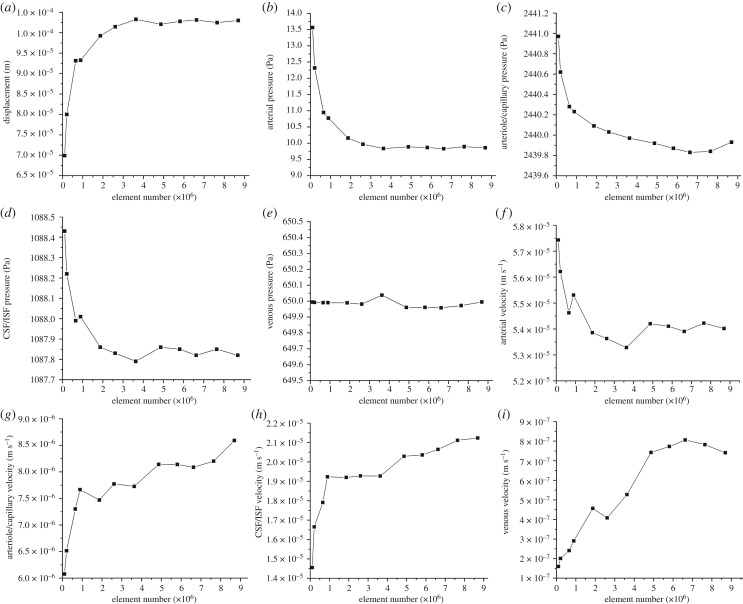
(*a*–*i*) Mean values of displacement magnitudes, pore pressures and Darcy velocities of the four fluid compartments in the parenchymal domain. These mean values are plotted against the number of tetrahedral elements (order of 10^6^).

In this study, all the simulations used the same set of parameters and BCs. The differences in the numerical solutions are caused purely by the varying mesh sizes. In the MPET model, the primitive variables are displacement and the scalar pore pressures of the four fluid compartments. Additional variables of practical interest, such as Darcy velocity and increment of fluid content [28], are derived from these primitive variables. The convergence of displacement magnitudes, pore pressures and Darcy velocities are shown in figure 4 (mean values in the entire parenchymal domain). It can be seen that all the variables are converging as the number of tetrahedral elements increases.

**Figure 5.**
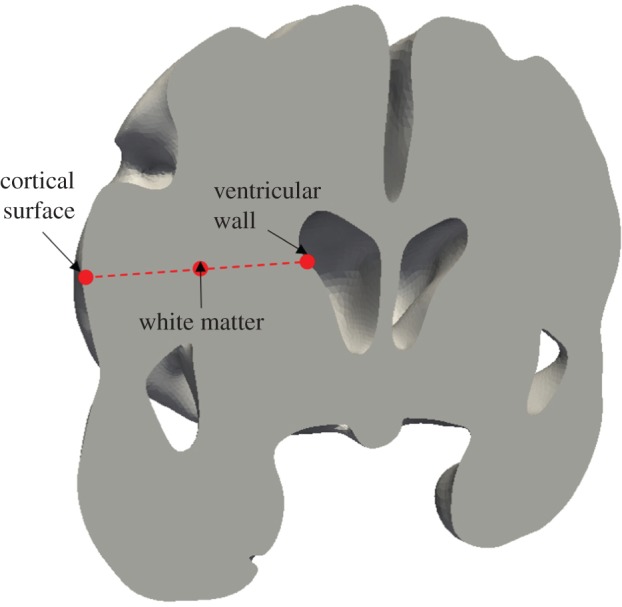
Selected positions in the parenchymal tissue domain to check local convergence behaviour with gradually refined meshes. (Online version in colour.)

In addition, three points were selected in the domain to show the local convergence behaviour at different representative positions, i.e. the white matter, the cortical surface and the ventricular wall (figure 5). The variables that are relevant to §4 of this paper, i.e. the parenchymal tissue displacement, the pore pressure of the CSF/ISF compartment, the Darcy velocities of the arteriole/capillary and the CSF/ISF compartments, are plotted against element number (table 3). It should be noted that for some variables, due to the application of Dirichlet BCs, the values at these boundaries remain constant with refined element sizes (not plotted). In §4, the pore pressure of the CSF/ISF compartment is referred to as the intracranial pressure (ICP), and the Darcy velocities of the arteriole/capillary and the CSF/ISF compartments are used to represent blood perfusion and CSF/ISF clearance. It can be seen from table 3 that these variables converge as the element number increases in three positions in the brain. Therefore, together with the results from figure 4, it is reasonable to suggest that for this level of brain geometry complexity, 2 million tetrahedral elements are sufficient to give convergent solutions. This criterion is satisfied in the subject-specific modelling, where the meshes have 2–3 million elements.

**Table 3.** Convergence plots as element number increases at different positions in the parenchymal domain. It should be noted that there are Dirichlet BCs applied for certain variables, so the values do not change as the element number increases and their plots are not included here.

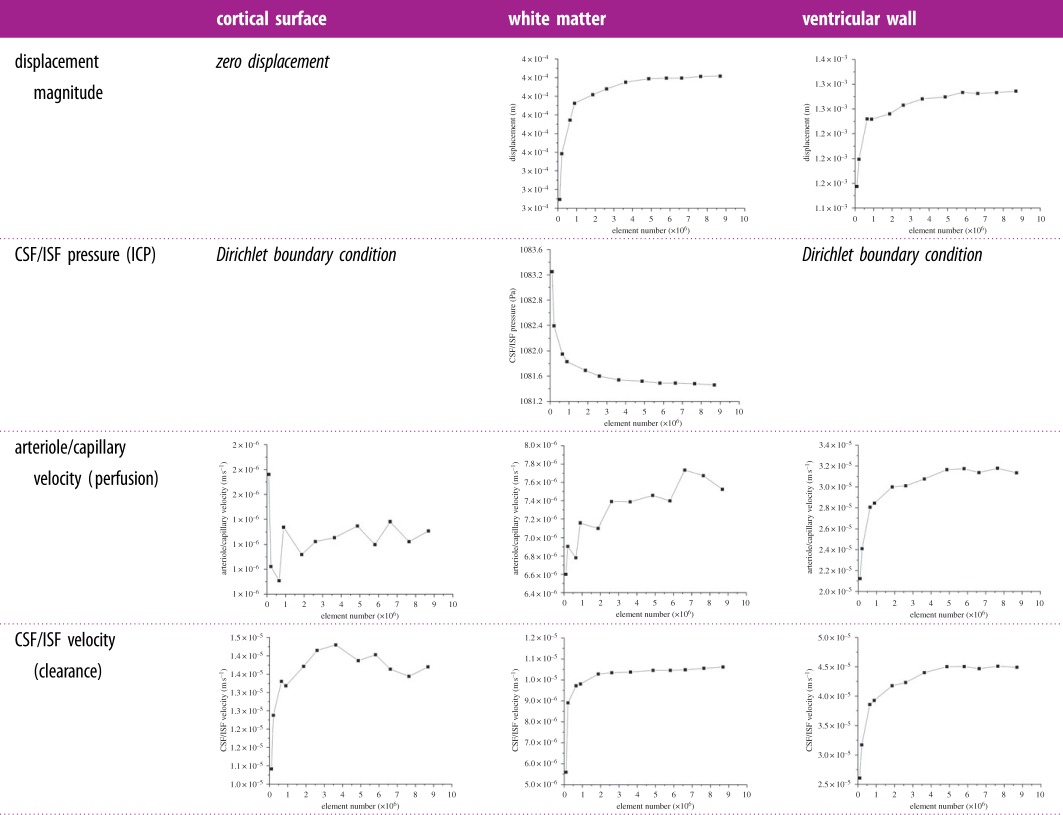

### 2.3. Subject-specific brain modelling pipeline

**Figure 6.**
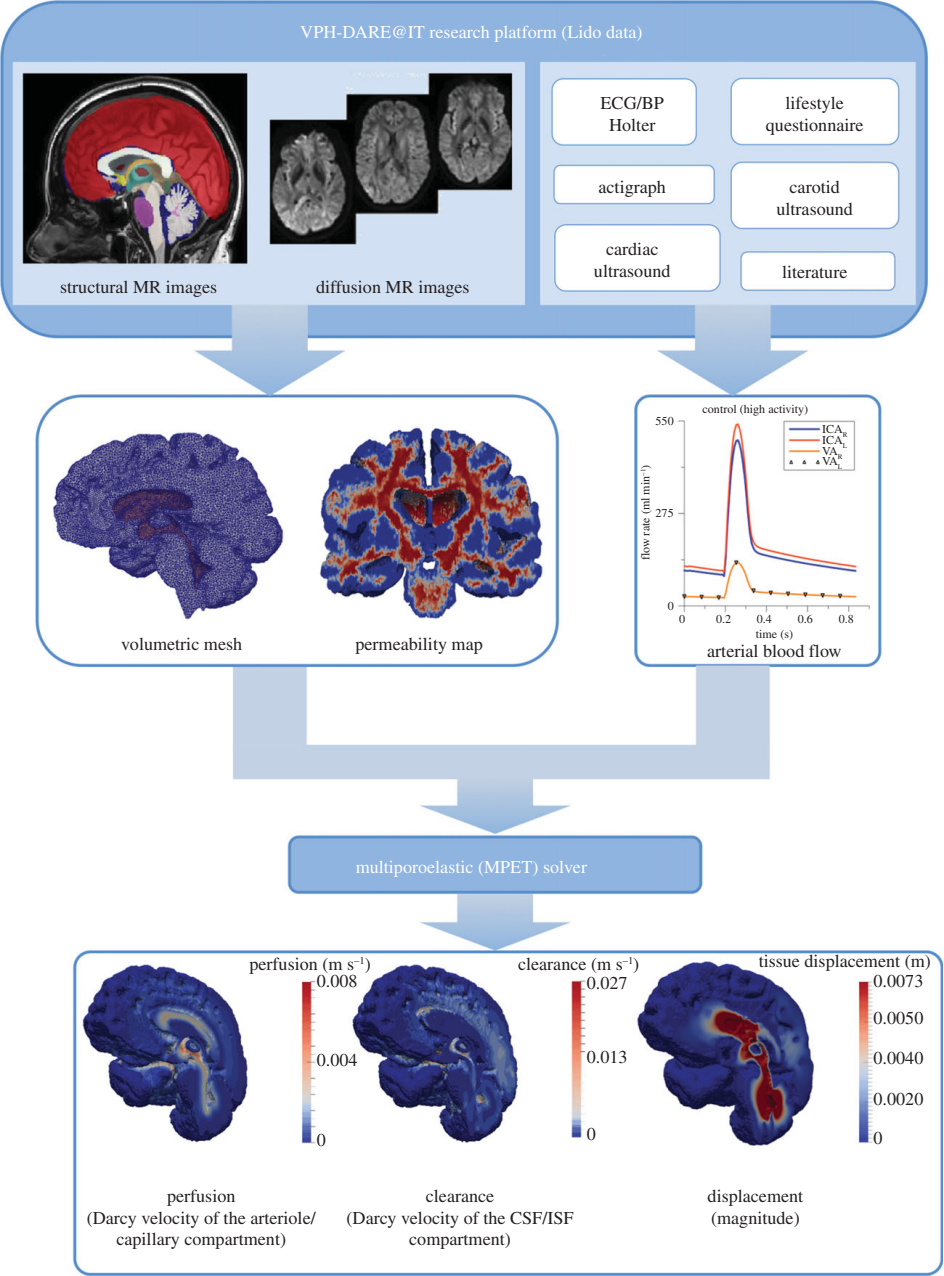
The consolidated pipeline that incorporates the 3D MPET solver itself, with image- and non-image-based model personalization modules. (Online version in colour.)

The MPET system gives rise to a generic model that simulates biomechanical behaviour of perfused tissue. The models presented here are personalized for individual subjects on three levels: cerebral geometries, and corresponding computational meshes, were extracted from structural MR images; spatial maps of CSF/ISF compartment permeability tensors were estimated from diffusion MR data; and arterial blood flow waveforms, used as cortical surface BCs, were derived from measurements of blood pressure, flow velocity and other inputs as described in §2.3.3. The consolidated pipeline (and its outputs) is depicted in figure 6, and its details will be described in the following three sections. It was implemented on the MULTIX platform, developed within the VPH-DARE@IT project. The latter provides a software infrastructure for integration and harmonization of disparate data inputs from multiple collections, and for orchestrating large-scale analyses of the same using cloud, and other, computing resources. It correspondingly streamlines the deployment of complex computational tool-chains, as described here, on data from large cohorts of subjects.

#### 2.3.1. Image-based construction of brain geometries and meshes

**Figure 7.**
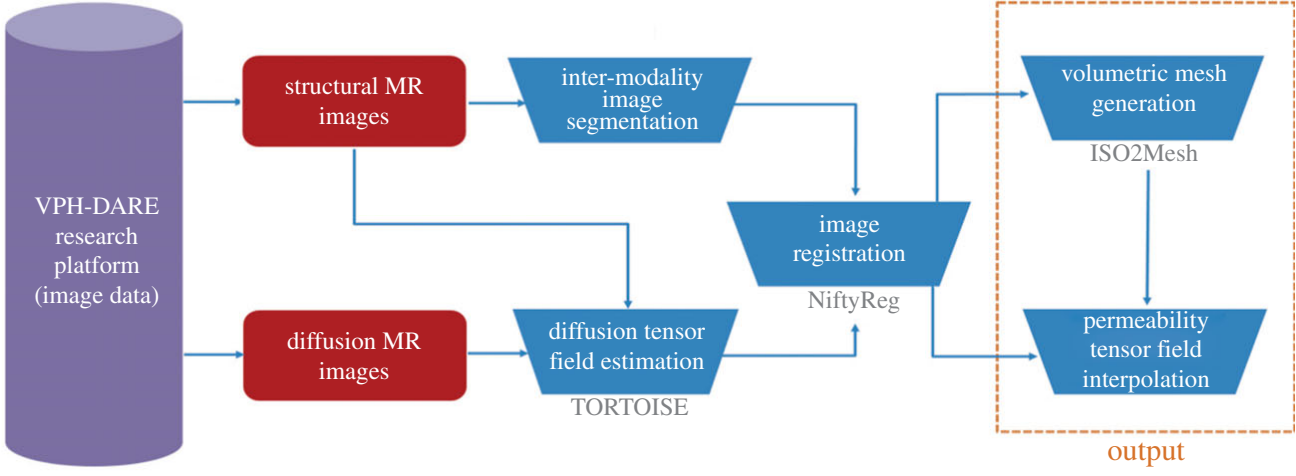
Workflow for the generation of subject-specific meshes and PTMs. The references for the tools used in this workflow can be found in [35] (TORTOISE), [36] (NiftyReg) and [37] (ISO2Mesh). (Online version in colour.)

**Figure 8.**
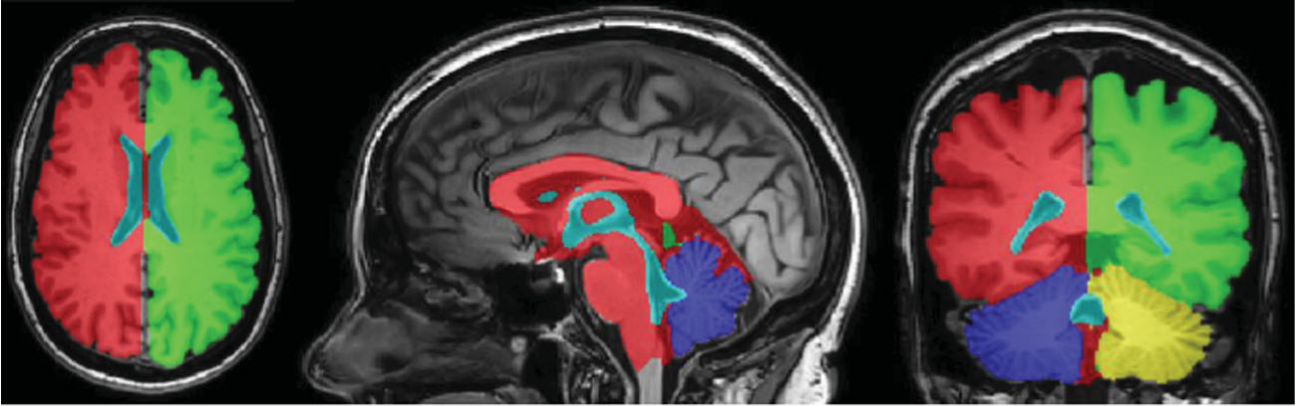
Region labels retained in segmentations. Distinct features incorporated into the model using the atlas-based propagation function, such as interthalamic adhesion, fourth ventricle and separation of cerebral hemispheres, are clearly visible. (Online version in colour.)

**Figure 9.**
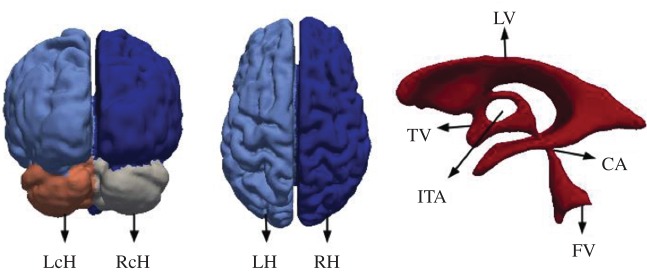
Brain geometry with distinct surface region labels, such as left cerebellar hemisphere (LcH), right cerebellar hemisphere (RcH), left cerebral hemisphere (LH), right cerebral hemisphere (RH); and the ventricular system comprising lateral ventricles (LV), third ventricle (TV), interthalamic adhesion (ITA), fourth ventricle (FV) and cerebral aqueduct (CA). (Online version in colour.)

**Figure 10.**
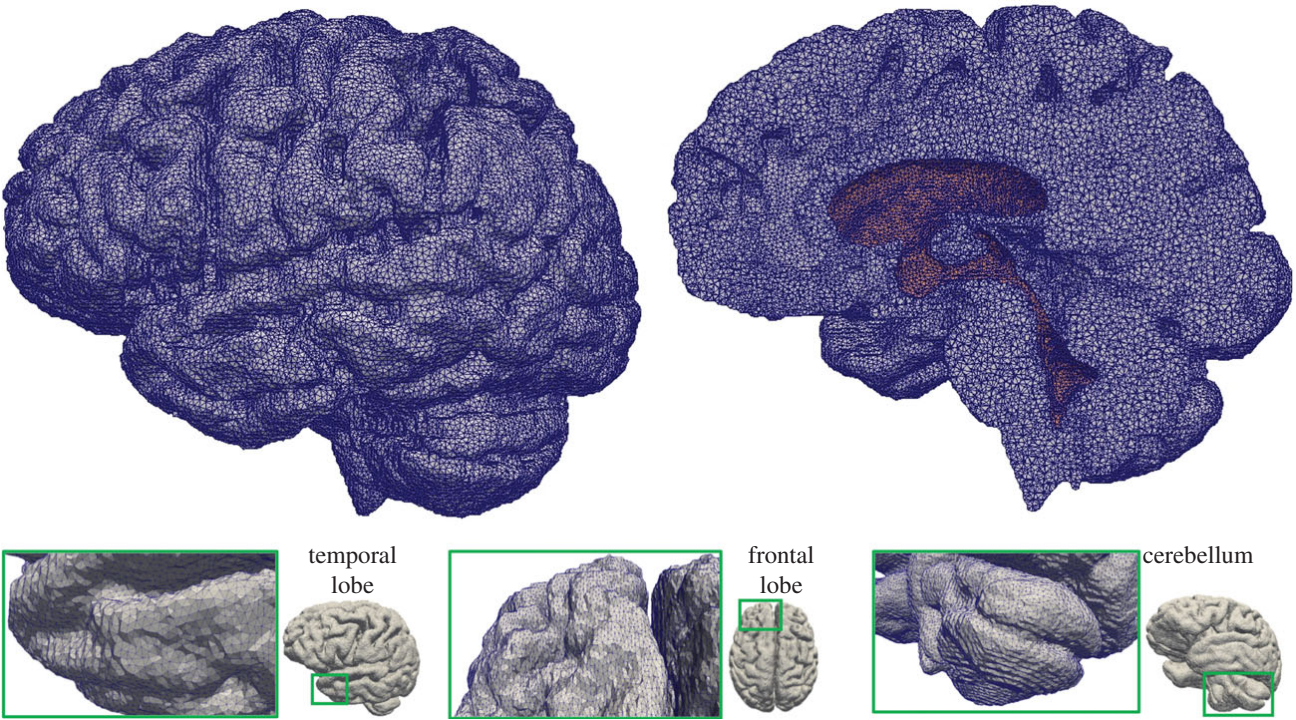
Tetrahedral mesh of the control subject generated from T1w-MR images. The figure on the top right-hand side shows a cross section of the volumetric mesh, where the dark area represents the ventricular wall. The number of tetrahedral elements in this case is 2 772 075. (Online version in colour.)

In the case of MPET modelling, two main components are needed from brain models: firstly, an accurate representation of the brain anatomy, with clear distinctions between cerebral and cerebellar hemispheres, as well as the ventricular system; secondly, the permeability at different regions of the brain tissue (see §2.3.2). A fully automated workflow (figure 7) has been developed to provide subject-specific meshes and PTMs for the MPET modelling. The implementation of this workflow used several algorithms, including both publicly available sources in addition to newly developed ones. The references for the tools used in this workflow can be found in [35] (TORTOISE), [36] (NiftyReg) and [37] (ISO2Mesh). The first step of this workflow is image segmentation, which features detailed subcortical (including the ventricular system) and whole-brain segmentation of T1w-MR images. Fully automatic segmentation of T1w-MR images was performed, and unique labels were retained only for the regions of interest for personalized mesh generation. Labels extracted from the generated segmentations, incorporating additional features such as separation of the cerebral hemispheres and the cerebroventricular system, are shown in figure 8. Sharp features, irregular surface geometries and disconnected island-like artefacts are addressed by smoothing the segmented images (figure 9). An acceptable balance, for the purpose of the MPET simulations, is struck between elimination of artefacts and retention of surface features. Following image segmentation, the next step is volumetric mesh generation. The workflow is able to create tetrahedral meshes of sufficient quality, which consequently preserves structural detail. Figure 10 portrays a uniform element size and distribution within brain parenchyma, and a higher mesh density in regions close to the cortical surface and the ventricular wall.

#### 2.3.2. Image-based modelling of cerebrospinal fluid/interstitial fluid compartment permeability

**Figure 11.**
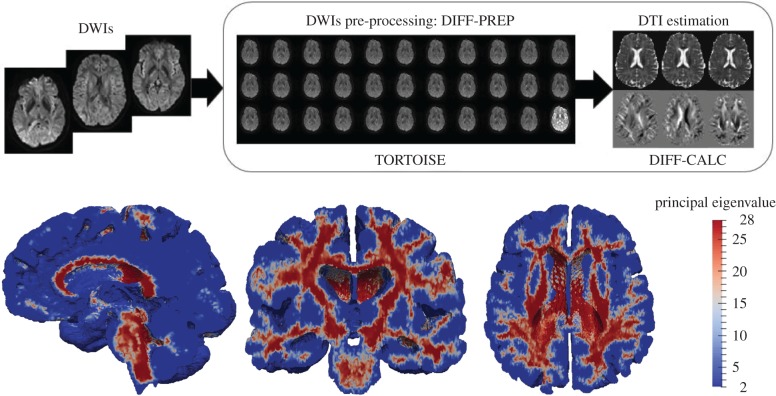
(Top row) Workflow depicting results from pre-processing DWIs and estimating the DT field using TORTOISE [35]. (Bottom row) Principal eigenvalues in the PTM of the control subject. (Online version in colour.)

The other important component from the output of this workflow (figure 7) is subject-specific PTMs. The estimated diffusion tensor (DT) field and its associated principal eigenvectors were used to estimate PTMs. The intermediate and final results from processing diffusion-weighted imaging (DWI) using TORTOISE [35] are summarized in figure 11. The permeability map gives one permeability tensor at each tetrahedral element, so in view of the entire brain, a heterogeneous and anisotropic permeability field has been captured. Figure 11 also shows the permeability map in terms of principal eigenvalues, which are plotted element-wise in three mesh cross sections. This PTM was used for the CSF/ISF compartment in the 3D MPET modelling.

#### 2.3.3. Boundary conditions for arterial blood flow

The remaining component of the subject-specific modelling pipeline is the personalized BCs for arterial blood flow. A subject-specific characterization of 24 h blood flow variability is obtained through a combination of ambulatory blood pressure measurements, clinical ultrasound flow measurements and mathematical modelling [38]. A lumped parameter circulation model (LPCM) [39] is used to simulate continuous arterial blood flow and translate spot measurements collected at 15 min intervals to continuous waveforms of arterial blood flow. Collected data are then used for model personalization, either as direct inputs (heart rate) or for optimizing the model parameters. In the latter case, measured quantities (systolic/diastolic blood pressure, left ventricle ejection fraction and end-diastolic volume) are used as a reference, and the model parameters are tuned with the goal of achieving the best possible fit of the experimental data. Once the arterial flow waveforms in the internal carotid artery (ICA) are obtained from the LPCM, they are coupled with another lumped parameter model for cerebral flow autoregulation [40]. The final output of this model is a 24 h prediction of middle cerebral artery flow, where a minimum baseline flow into the brain is preserved by a simple two-element feedback control model. Finally, these continuous waveforms are fed into the MPET modelling as BCs for the arterial compartment at the cortical surface.

**Figure 12.**
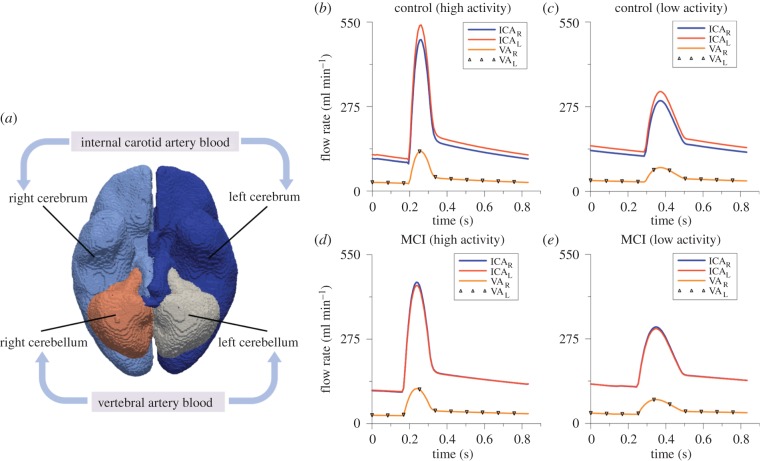
(*a*) The four regions on the cortical surface that are used for the personalized BCs of arterial blood flow. (*b*) A snapshot of the waveforms of left and right ICA and VA flow profiles corresponding to the period of high activity within a 24 h period for the control subject, (*c*) low activity for the control subject, (*d*) for high activity for the MCI subject and (*e*) for low activity for the MCI subject. (Online version in colour.)

For each subject, four waveforms were calculated at every time point, which are the ICA blood to the left and right cerebrum (ICA_L_ and ICA_R_), and the vertebral artery (VA) blood to the left and right cerebellum (VA_L_ and VA_R_). In order to apply these subject-specific waveforms as BCs for the arterial compartment, the cortical surface is divided into four perfusion regions corresponding to the four waveforms (figure 12*a*). The region labels are propagated through the segmentation tool. In the 3D MPET model, the surface area of each region is calculated in the pre-processing. The total amount of arterial blood flow is distributed across each perfusion region and applied as a flow BC to the MPET model. This is a simplification of the true cerebral arterial perfusion network of anterior/middle/posterior cerebral arteries extending along the pial surface before dividing into smaller penetrating arteries and arterioles that perfuse the cerebral cortex and deep white matter.

The subject-specific BCs of arterial blood flow were recorded every 15 min during the day and every 30 min during the night. Here, two specific measurements were chosen from the 24 h recording as indicative of the subject's activity—the first one is high activity (e.g. exercise) identified by the highest peak values of arterial blood flow within 24 h; and the second one is low activity (e.g. sleep) identified by the lowest peak values. The waveforms corresponding to these two activity states were used in the subject-specific modelling of this paper as BCs for the arterial compartment (figure 12*b*–*e*).

## 3. Results

Figure 12 depicts subject (and activity) specific profiles of ICA and VA flow in both left and right sides of the cerebrum and cerebellum. The arterial feeding territories are the left/right cerebrum (using the ICA_L/R_ profiles) and the left/right cerebellum (using the VA_L/R_ profiles). Figure 12*b*,*c* shows the profiles corresponding to a period of high and low activity for the control subject. Figure 12*d*,*e* shows the same profiles corresponding to a period of high and low activity for the MCI case. The peak flow rate is extracted from each of these curves, and the results are listed in table 4. The left ICA possesses higher flow rates for the control case during both high and low activity. For the MCI case, the flow rate is higher in the right ICA during high and low activity.

**Table 4.** Peak flow rate during high and low activity corresponding to the control and MCI cases.

	flow rate (ml min^−1^)					
	high			low		
	ICA_L	ICA_R	VA_L/R	ICA_L	ICA_R	VA_L/R
control	540.6	493.1	129.2	324.3	294.5	77.3
MCI	449.9	460.2	113.8	308.1	313.7	77.7

Table 5 shows the results associated with the difference between maximum and minimum arterial blood pressure (ΔABP) and ICP (ΔICP) values in the left (L) and right (R) hemispheres. Both control and MCI results showed a reduction in ΔABP and ΔICP between the two activity states. The control case exhibited a reduction of under 1 mmHg in ΔABP for both hemispheres, while the MCI case showed signs of asymmetric reduction in ΔABP, with values of approximately 1.6 and 1.4 mmHg for left and right hemispheres, respectively. As for the ΔICP, the control and MCI cases exhibited a reduction of approximately 0.5 and 0.4 mmHg between high and low activity states.

**Table 5.** Range of ABP and ICP (max–min) for each hemisphere during high and low activity corresponding to the control and MCI cases.

	ΔICP (mmHg)				ΔABP (mmHg)			
	high		low		high		low	
	L	R	L	R	L	R	L	R
control	2.46	2.46	1.98	1.98	12.22	12.08	11.25	11.10
MCI	2.16	2.16	1.75	1.75	12.45	10.28	10.88	8.85

**Figure 13.**
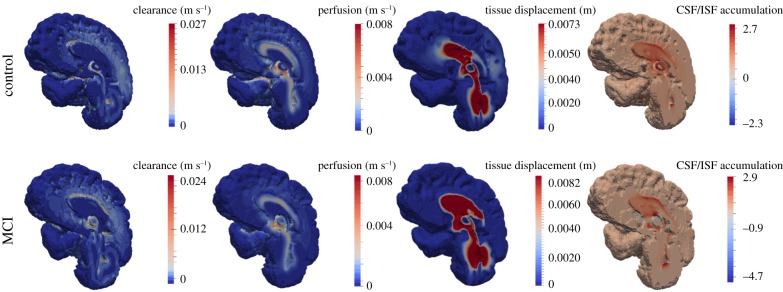
Midsagittal section depicting MPET results for CSF/ISF clearance, blood perfusion, parenchymal tissue displacement and accumulation of CSF/ISF. All results are acquired during a period of high activity. (Online version in colour.)

Figure 13 depicts four solution fields arising from the MPET solver, namely clearance (Darcy velocity of CSF/ISF compartment), blood perfusion (Darcy velocity of capillary compartment), parenchymal tissue displacement and CSF/ISF accumulation (positive values of fluid content, *ζ*_e_, of the CSF/ISF compartment). Comparing the results between the control and MCI case, it can be seen that parenchymal tissue displacement and CSF/ISF drainage (*ζ*_e_ < 0) are more pronounced. Blood perfusion between the two cases remains at roughly similar levels, peaking at 8.0 mm s^−1^. The peak clearance in the MCI case is marginally smaller, at 2.4 cm s^−1^ in the vicinity of the lateral ventricle. For the simulations relating to low activity, the two sets of results (control and MCI) for the variables of interest are: peak clearance (1.6 and 1.7 cm s^−1^), peak perfusion (7.3 and 6.6 mm s^−1^), peak tissue displacement (7.4 and 8.2 mm), peak CSF/ISF accumulation (2.9 for both cases) and finally CSF/ISF drainage (−2.3 and −4.7).

**Figure 14.**
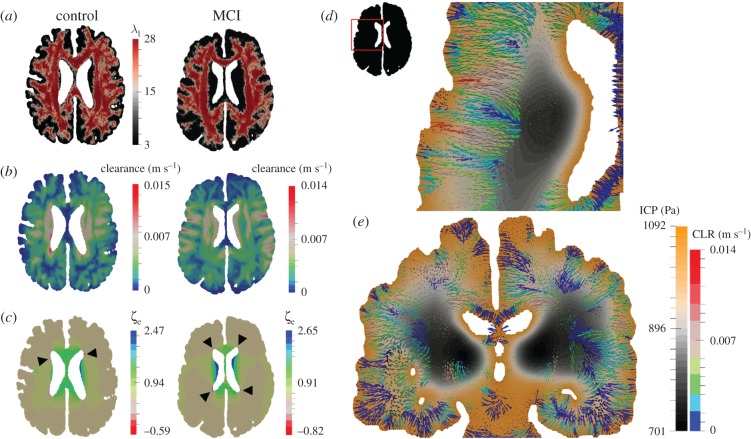
Axial slices through the cases corresponding to a male 66-year-old control and a male 78-year-old with MCI. (*a*) Principal eigenvalue of the permeability tensor, (*b*) clearance and (*c*) CSF/ISF accumulation (*ζ*_e_). The two latter sets of results depict the influence of incorporating anisotropic permeability (which is influenced by the local microstructure). Black arrows indicate the regions with peak drainage for the respective cases. (*d*) An axial slice of the MCI case depicting the clearance (CLR) of CSF/ISF (in the form of filtration velocity vectors) superimposed with the ICP solution field. (*e*) A coronal slice of the MCI case depicting clearance (CLR) of CSF/ISF superimposed with the ICP solution field. (Online version in colour.)

Figure 14*a*–*c* gives a detailed representation of the interlinked nature of microstructural organization (white matter) within the parenchyma (principal eigenvalue map of the permeability tensor for the slice) and the solution fields relating to CSF/ISF clearance and accumulation. Observing figure 14*b*, CSF/ISF clearance shows an increased magnitude for the control case; however, the solution field characteristics are not similar. This is also true of the extent of CSF/ISF accumulation and drainage. For the latter, the focal points are the periventricular areas of high concavity on the anterior and posterior horns of the lateral ventricles. Arrows indicate the regions with peak drainage for the respective cases. Figure 14*d*,*e* shows two overlapping solution fields relating to the MCI patient, that of clearance and ICP.

## 4. Discussion

In this work, the emphasis has been placed on being able to associate some of the resulting outputs of the consolidated framework outlined in §2 with a potential set-up to acquire AD-specific biomarkers.

### 4.1. ΔArterial blood pressure, Δintracranial pressure and flow rate

From figure 12 and the constituent peak values in table 4, it can be observed that there is an increased blood flow rate from the ICA (left and right) and VA during the period of high activity when comparing the control and MCI case. The lower flow rate in the ICA and VA (that could be theoretically linked to a reduced cardiac output) associated with the MCI case may hint to a subtle underlying hypoperfusion that could develop further in this patient. The results presented here compare well with resting state values observed in the literature between control and MCI cohorts [41,42]. It can be postulated that chronically reducing cardiac output can be damaging, as studies have shown that cerebral autoregulation does not necessarily protect the brain under these circumstances [17]. This ultimately affects the maintenance of cerebral perfusion pressure during ageing, which may subsequently promote early glial activation, damage or destroy neurons in addition to affecting necessary glucose distribution [17,43–45]. Various cohort and population studies have shown an association between hypertension and cognitive impairment [46], and it is a well-known risk factor for developing dementia [1,47]. The results in table 5 indicate the ΔABP and ΔICP in both hemispheres during both high and low activity. There was a reduction in ΔABP of less than 1 mmHg for both hemispheres in the control case, while the MCI case produced an asymmetric reduction in ΔABP (greater than 1 mmHg). The variation in ΔICP between activity states was not as pronounced. This is anticipated, owing to the reduced flow rates during low activity that are prescribed as arterial BCs to the partitioned cortical surface shown in figure 12, and the subsequent need for the arterial pore pressure solution fields to impose themselves through the source terms in the CSF/ISF compartment (equation (2.4)). An intrinsic requirement is that the arteriole/capillary compartment acts as a mediator to this process (owing to the directional constraints between compartments (figure 1 and [48]).

### 4.2. Clearance, perfusion, cerebrospinal fluid accumulation and parenchymal tissue displacement

Figure 13 shows that applying the aforementioned subject-specific BCs to the arterial compartment influences the morphology of the solution fields of the remaining MPET fluid compartments, in addition to the magnitude of displacement of the parenchymal tissue (**u**). Global clearance is marginally lower for the MCI case, while tissue displacement and CSF/ISF accumulation (and drainage) are both increased. The level of perfusion is similar (peak perfusion of 8 mm s^−1^); however, the solution field differs substantially in spatial morphology. Aβ homeostasis is governed by production and clearance mechanisms [3]. Any subsequent imbalance in this homeostasis may result in excessive accumulation of cerebral Aβ, which is a known characteristic of AD [15]. Evidence suggests that impaired Aβ clearance is apparent in both early- and late-onset forms of AD [2]. Considering the inherent need to critically understand the mechanism behind Aβ clearance, the current pipeline presented in this paper can be considered as a possible test-bed for various hypotheses, such as the influence of continuity between the ventricular system, cisterns, para-vascular spaces and the subarachnoid space (SAS) [49,50]. Soluble Aβ can be transported via the following clearance mechanisms [2]: across the blood–brain barrier (BBB) and blood–CSF barriers, ISF bulk flow (facilitated by astroglial aquaporin-4 channels) and CSF reabsorption into the circulatory and lymphatic systems, and finally enzymatic degradation and cellular uptake. In figure 13, the clearance mechanism responsible for the results shown can be deemed to possess an overlapping nature between BBB and ISF bulk flow mediated uptake, because the arteriole/capillary and CSF/ISF compartments are interlinked via the relevant spatially varying source densities.

The results relating to CSF/ISF accumulation give an indication of the degree of swelling within the parenchymal tissue [28]. It is evident that periventricular swelling dominates the solution field, while there are strategically located pockets of extensive CSF/ISF extravasation (*ζ*_e_ < 0). Similar solution fields are obtained for the same cases during low activity (sleep), and the results associated with figure 13 are mentioned in §3 for reference. The tightly coupled nature between the arterial BCs and the remaining compartments of the MPET system is observed, considering there is an overall global reduction in CSF/ISF clearance and blood perfusion during the simulations involving the flow profiles originating from low activity. Both **u** and *ζ*_e_ remain largely unaffected during this reduction in arterial blood flow rate.

The anisotropic nature of CSF/ISF fluid transport within the brain is accounted for via the subject-specific tissue permeability maps estimated from DT fields. These are encoded within the tetrahedral element-based meshes used to conduct the finite-element-based MPET simulations. Tuch *et al*. [51] and Sarntinoranont *et al*. [52] presented evidence that both the DT and permeability tensors share the same principal eigenvector. Periventricular lucency [53] is represented by positive *ζ*_e_ values in the periventricular regions (qualitative agreement exists in [28]). It is assumed to derive from ependymal surface breakdown, which helps alleviate some of the pressure in the cerebroventricular system by allowing for some CSF extravasation (facilitated by aquaporin-4 [50,54]).

It can be seen from figure 14*a*–*c* that the underlying variations in directionality imposed by the permeability tensor strongly influence the clearance pattern between the surface of the ventricles and cortices. For the drainage of CSF/ISF (*ζ*_e_ < 0), the areas with peak values are highlighted in the figure. It is observed that these areas largely coincide with areas of high clearance, and support the CSF/ISF extravasation theory in these locations (pore pressure is positive in these areas [55]). The latter solution field is also influenced by the strain alterations within the parenchymal tissue [55], and to a lesser extent, the weighted proportion of ICP [28]. Further refinement in this component of the pipeline may provide further justification of the association between surrogate markers of brain dysfunction, such as in white matter lesions with cognitive impairment and dementia [56]. From figure 14*d*,*e*, it can be seen that the nature of the spatial distribution of CSF/ISF clearance within the parenchyma is not entirely dependent on the local underlying microstructure, but also on the ICP (which lies within physiological levels [57]). Interrogating the differences in mechanical loads experienced by the brain and the ensuing differences in fluid clearance could identify new biomarkers related to vascular risk factors of AD.

### 4.3. Limitations and future work

In this extensive, yet preliminary study, only two non-smokers from the Lido study cohort were analysed (execution of the pipeline for the remaining cases is currently in progress). In addition, the control subject lacked relevant information related to their sleeping pattern (sleep-correlated changes of cerebral blood flow, clearance of ISF etc. could not be made). It would therefore be premature to expect the current results to confirm the veracity of any association between these important risk factors and cognitive impairment. The consolidated pipeline presented in this work is able to integrate principles of solid and fluid mechanics (as in [22,28]), and may aid in better understanding hypotheses such as those identifying the intricate mechanisms relating to pressure-driven atrophy within the parenchymal tissue [58], in addition to considering the role of repetitive and intensifying arterial pulsations (due to the increased stiffness of the arterial tree during ageing) on the underlying neuronal microstructure and glial function [16,58]. The link between modifiable LFs and cognitive decline is complex. In the future, the influence of LFs that have an effect on the vascular (cardiovascular and neurovascular) system physiology will be investigated (such as smoking). Modelling an LF by personalizing systemic variables, BCs and tissue constitutive parameters can allow for an extended pipeline which may account for the long-term response to hypothetical LF patterns and timings.

Hypertension may itself result in hypoperfusion, via remodelling of small blood vessels and white matter alterations [1]. The MPET modelling framework is capable of incorporating these features, provided that additional properties associated with cognitive impairment are accounted for, such as the expected variation in mechanical tissue properties and vascular compliance. Importantly, this will form part of a broader study, which will involve the assessment of the consolidated pipeline using the 103 Lido datasets.

The higher than usual ICP pressure gradients (typically, only small fluctuations are observed in the literature [59]) are due to the blood and CSF/ISF compartments of the MPET system being in tight communication via their intercommunicating spatially varying source/sink terms. These terms require further work in order to better accommodate physiologically representative microscale fluid transport. Recent simulations of interstitial bulk flow within 3D electron microscope reconstructions of hippocampal tissue [59] should also be used as a means of conceptually extending the MPET model (for instance, adding additional compartments in order to simulate the CSF and ISF compartments separately) in order to more accurately account for the glymphatic system [60], and the understanding around its key constituents (interstitial solute transport) [48]. Recent studies have shown that solutes of varying sizes can be more easily transported through the interstitium via diffusion as opposed to bulk flow, and when combined with para-vascular advection, can provide a credible alternative to the lymphatic drainage systems found in other organs [59].

The cortical SAS is neglected in the current study. Its inclusion would allow for the relaxing of the assumed rigid BC currently applied to the cortical surface. This would allow for more accurate results relating to global parenchymal tissue displacement (over the course of one or more cardiac cycles), in addition to enhancing our understanding of intracranial dynamics when the MPET model is extended to include fluid–structure interaction.

Finally, the set of continuity equations for the MPET system should take cross-porosity storage effects into account [61–63]. Further limitations associated with the MPET system have already been previously outlined [28].

## 5. Conclusion

This paper introduces a consolidated pipeline that intertwines a 3D multi-poroelastic model of parenchymal tissue, an image-based modelling pipeline and a detailed subject-specific BC model. The implementation of the pipeline was described, and is highlighted as the key component of this paper. Two cases were simulated, one involving a 66-year-old male control subject and one involving a 78-year-old male MCI case. Both of the cases were prescribed arterial BCs relating to a period of high and low activity.

## Data accessibility

This article has no additional data.
